# Depression, anxiety and major adverse cardiovascular and cerebrovascular events in patients following coronary artery bypass graft surgery: a five year longitudinal cohort study

**DOI:** 10.1186/s13030-015-0041-5

**Published:** 2015-05-26

**Authors:** Phillip J. Tully, Helen R. Winefield, Robert A. Baker, Johan Denollet, Susanne S. Pedersen, Gary A. Wittert, Deborah A. Turnbull

**Affiliations:** Department of Rehabilitation Psychology and Psychotherapy, Institute of Psychology, University of Freiburg, Engelbergstr. 41, D-79085 Freiburg, Germany; Freemasons Foundation Centre for Men’s Health, Discipline of Medicine, School of Medicine, The University of Adelaide, Adelaide, Australia; Department of Medicine, Cardiac Surgery Research, Department of Surgery, School of Medicine, Flinders University of South Australia, Adelaide, Australia; School of Psychology, The University of Adelaide, Adelaide, Australia; CoRPS, Center of Research on Psychology in Somatic Diseases, Tilburg University, Tilburg, The Netherlands; Department of Psychology, University of Southern Denmark, Odense, Denmark; Department of Cardiology, Odense University Hospital, Odense, Denmark; Department of Cardiology, Thoraxcenter, Erasmus Medical Center, Rotterdam, The Netherlands

**Keywords:** Coronary artery bypass grafts, Coronary heart disease, Depression, Generalized anxiety disorder, Prognosis, Survival analysis, Cardiovascular disease

## Abstract

**Background:**

Although depression and anxiety have been implicated in risk for major adverse cardiovascular and cerebrovascular events (MACCE), a theoretical approach to identifying such putative links is lacking. The objective of this study was to examine the association between theoretical conceptualisations of depression and anxiety with MACCE at the diagnostic and symptom dimension level.

**Methods:**

Before coronary artery bypass graft (CABG) surgery, patients (N = 158; 20.9 % female) underwent a structured clinical interview to determine caseness for depression and anxiety disorders. Depression and anxiety disorders were arranged into the distress cluster (major depression, dysthymia, generalized anxiety disorder, post-traumatic stress disorder) and fear cluster (panic disorder, agoraphobia, social phobia). Patients also completed the self-report Mood and Anxiety Symptom Questionnaire, measuring anhedonia, anxious arousal and general distress/negative affect symptom dimensions. Incident MACCE was defined as fatal or non-fatal; myocardial infarction, unstable angina pectoris, repeat revascularization, heart failure, sustained arrhythmia, stroke or cerebrovascular accident, left ventricular failure and mortality due to cardiac causes. Time-to-MACCE was determined by hazard modelling after adjustment for EuroSCORE, smoking, body mass index, hypertension, heart failure and peripheral vascular disease.

**Results:**

In the total sample, there were 698 cumulative person years of survival for analysis with a median follow-up of 4.6 years (interquartile range 4.2 to 5.2 years) and 37 MACCE (23.4 % of total). After covariate adjustment, generalized anxiety disorder was associated with MACCE (hazard ratio [HR] = 2.79, 95 % confidence interval [CI] 1.00-7.80, *p* = 0.049). The distress disorders were not significantly associated with MACCE risk (HR = 2.14; 95 % CI .92-4.95, *p* = 0.077) and neither were the fear-disorders (HR = 0.24, 95 % CI .05-1.20, *p* = 0.083). None of the symptom dimensions were significantly associated with MACCE.

**Conclusions:**

Generalized anxiety disorder was significantly associated with MACCE at follow-up after CABG surgery. The findings encourage further research pertaining to generalized anxiety disorder, and theoretical conceptualizations of depression, general distress and anxiety in persons undergoing CABG surgery.

## Introduction

Depression dominates recent understandings of the putative links between negative emotions and major adverse cardiovascular and cerebrovascular events (MACCE, e.g. myocardial infarction, stroke) in patients with coronary heart disease (CHD) [1]. However, despite depression treatment with psychotherapy and antidepressant interventions [2, 3], a consistent reduction in MACCE remains elusive in the population with comorbid depression and CHD [3], raising questions about the focus of interventions [4]. Indeed, depression is but one of a spectrum of disorders (e.g. anxiety, post-traumatic stress disorder and panic disorder) which are purported to deleteriously affect CHD outcomes [5–9]. In fact, most negative emotional risk factors for MACCE share a common predisposition to negative affectivity (NA), also known as neuroticism, evident at both the theoretical [10, 11] and measurement level [12–14]. Past attempts to clarify the risk of MACCE attributable to depression independent of NA and anxiety have not been consistently supported [12, 15–19], and a theoretical approach to this field is lacking [13]. Therefore the aim of this study is to utilize theoretical conceptualizations of depression, NA and anxiety at the diagnostic disorder, cluster and symptom level. Such theoretical conceptualizations will, in turn, be employed to predict the prognostic MACCE outcome in a sample of patients undergoing coronary artery bypass graft (CABG) surgery.

Contemporary understandings of psychiatric nomenclature indicate that NA is the most ubiquitous feature of depression and anxiety disorders [10, 20–23]. Beyond the NA commonality, recent research also shows that certain depression and anxiety disorders relate more strongly to each other, and do not necessarily fall within prescribed diagnostic categories of anxiety and depression. Specifically, previous empirical work largely supports a theoretical model with at least two groups of affective disorders. The first is collectively labelled the distress disorders and is comprised by major depression, dysthymia, post-traumatic stress disorder and generalized anxiety disorder (GAD) [10, 20–24]. Research also supports that symptoms of anhedonia are a dimensional marker for the distress disorders, and therefore, not only for major depression [10]. The second group of disorders are collectively labeled the fear disorders which is comprised of panic disorder, agoraphobia and social phobia [10, 20–24]. Symptoms of anxious arousal are a dimensional marker for the fear disorders [10], and therefore, not for other traditional anxiety disorders such as obsessive-compulsive disorder and post-traumatic stress disorder.

Along these lines, prior research in CHD populations has most commonly examined uni-polar and dysthymic depression subtypes [25–27] and anhedonia in relation to MACCE [28–31]. Also, some evidence in CHD populations suggests that post-traumatic stress disorder is associated with MACCE recurrence [32]. By contrast, GAD findings are more mixed [33–35] and there are too few studies on other anxiety disorder subtypes [36]. We are only aware of one study that assess anhedonia contemporaneously with anxious arousal and NA dimensions of the distress and fear clusters [37]. With these limitations in mind, we sought to extend the analysis of MACCE to 4.6 year follow-up. Based on the predominant research to date, we hypothesized that the distress disorder cluster and major depression especially would be significantly associated with MACCE at follow-up. Secondly, we hypothesized that the anhedonia dimension would be significantly associated with MACCE at follow-up.

## Method

### Patients

**Fig. 1:**
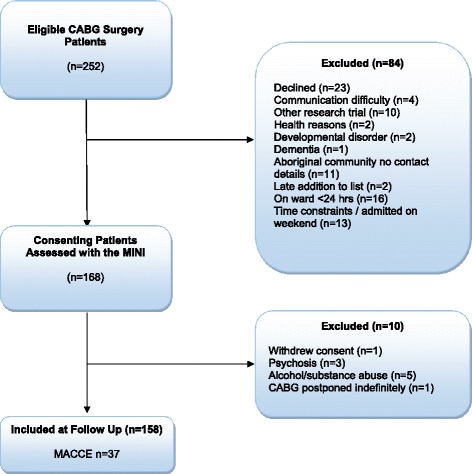
Flow chart of participants through the study. Graph showing selection and attrition of participants through the study. CABG, coronary artery bypass graft surgery; MACCE, major adverse cardiovascular and cerebrovascular events; MINI, MINI International Psychiatric Interview

Informed consent and ethics approval was obtained for this study (The University of Adelaide Human Research Ethics Committee approval # H-010-2007, The Flinders Medical Centre Human Research Ethics Committee approval 112/067) and the methods have been reported previously [37, 38]. Briefly, recruitment took place at the Flinders Medical Centre, South Australia, between February 2007 and March 2009. Patients were considered eligible if aged ≥ 18 years and undergoing CABG with cardiopulmonary bypass, and with or without concomitant valve procedures. From 252 eligible patients 84 patients were ineligible: declined (n = 23), communication difficulty (n = 4), participating in another research trial (n = 10), health reasons (n = 2), developmental disorder (n = 2), dementia (n = 1), living in a remote community with no contact details (n = 11), late addition to surgical list (n = 2), on the hospital ward <24 h (n = 16), time constraints/admitted on weekend (n = 13). From 252 eligible patients we recruited 168 and 10 patients were excluded further: surgery postponed indefinitely (n = 1), withdrawal of consent (n = 1), current psychosis and/or taking anti-psychotic medications (n = 3), current or past alcohol and/ or substance abuse (n = 5). This left a total sample of 158 patients (63 % participation rate). A flow chart of participants through the study is shown in Fig. 1. Non-respondents and excluded patients were more likely to identify as Aboriginal or Torres Strait Islander χ^2^ (1) = 5.85, *p* = .02 but were otherwise not discrepant from participants on baseline demographic and comorbid conditions. Medical data were prospectively collected by medical officers at pre-surgical consultation and entered directly onto an electronic database with quality assurance maintained at weekly database meetings by the third author. Data definitions utilize those of the Australian Society of Cardiac and Thoracic Surgeons [39] including permanent stroke, cerebrovascular accident or central neurological deficit persisting for longer than 72 h, myocardial infarction (2 or more of: enzyme level elevation; new wall motion abnormalities; serial EGG showing new Q waves).

### Structured interview

Patients were assessed a median of 3 days preoperatively (interquartile range 1–3 days). The MINI International Neuropsychiatric Interview (MINI) 5.0.0 [40, 41] was employed to determine psychiatric disorders by an intern psychologist (first author, with 1,000 h clinical psychology experience, employed 0.4 full-time equivalent in the study hospital). The MINI has high sensitivity and specificity to detect current mood and anxiety disorders, with Kappa coefficients (κ = .86 - .96) suggesting favorable agreement with Diagnostic and Statistical manual of Mental Disorders-IV (DSM-IV) [40, 42]. The MINI hierarchical diagnostic criteria stipulate that a GAD diagnosis cannot be made with a concurrent major depression diagnosis thus precluding comorbidity of these disorders and providing the advantage of classifying only primary affective disorders. Disorders were arranged into the distress cluster (major depression, dysthymia, post-traumatic stress disorder and GAD) and the fear disorder cluster (panic disorder, agoraphobia, and social phobia). We also considered affective disorders with more than 10 % prevalence at baseline as candidates for analysis in relation to MACCE.

### Self-report sistress assessments

#### Mood and anxiety symptom questionnaire

The self-report mood and anxiety symptom questionnaire (MASQ) was used to measure anhedonia, anxious arousal and general NA [43]. Based on the work of Wardenaar et al. [44, 45], we constructed a 30-item short form where 10-items each are allocated to an anxious arousal, anhedonia/low positive affect and general NA scale. Example items of anxious arousal include *“Was trembling or shaking”; “Had hot or cold spells”*. The anhedonic depression scale utilizes reverse-keyed items assessing positive emotional experiences including *“Felt like I had a lot to look forward to”; “Felt like I was having a lot of fun”*. Example items of the MASQ-general NA scale include *“Felt irritable”; “Worried a lot about things”*. The MASQ has been found to fit the three-dimensional model (general NA, anhedonia, anxious arousal) indicating good construct validity with high discriminant validity [44], and was recently validated in the English language [46]. Previous psychometric research with the MASQ has established good psychometric properties of this questionnaire [44, 46–52]. In the present sample satisfactory internal consistency was observed (Cronbach’s alpha coefficients; general NA = 0.88; anhedonia = 0.84; anxious arousal = 0.77).

### Major adverse cardiovascular and cerebrovascular events

The Australian Institute of Health and Welfare’s National Death Index was utilized to determine mortality data until the study census date 31st December 2013, according to the International Classification of Diseases (ICD) 10th Revision codes [53]. Hospital admission after discharge from the index CABG procedure was ascertained from patient medical records and electronic admission data linkage between hospitals in ICD code [53]. The MACCE endpoint was defined as fatal or non-fatal hospitalisation for myocardial infarction, unstable angina pectoris, repeat revascularization, sustained arrhythmia, stroke or cerebrovascular accident, heart failure, left ventricular failure and mortality due to cardiac causes, as consistent with previous research [54]. In this manner, any non-cardiac death is censored from the analyses at the date of death. All MACCE were inspected blinded to psychological distress scores.

### Statistical analysis

Data analysis was performed with SPSS® 22.0 statistical software package (SPSS Inc., Chicago, IL). Descriptive comparisons were made with the t-test, the chi-square test or the Fisher’s exact test as appropriate. All statistical tests were two-tailed, an alpha value *p* ≤ .05 was considered statistically significant, and no adjustment was made for multiple comparisons based on the recommendations of Rothman [55]. Adjusted Cox proportional model hazard ratios (HR) and 95 % confidence intervals (CIs) were used to determine the risk of MACCE associated with negative emotions. Candidate covariates for hazard models were selected *a priori* based on the literature to cover the covariates that are associated with depression and anxiety (independent variables) [56]. We also selected covariates associated with MACCE risk and cardiac surgery morbidity outcomes (dependent variables) [57, 58]. The candidate covariates included smoking, body mass index, hypertension, heart failure, peripheral vascular disease, and the European System for Cardiac Operative Risk Evaluation [59] (EuroSCORE). The EuroSCORE is calculated from 17 risk factors including age, sex, left ventricular dysfunction, previous cardiac surgery, elevated creatinine and concomitant procedures among others, and is associated with MACCE and survival in the long-term [57, 58]. The proportionality of hazards assumption was checked initially by entering covariates as interactions with time and also ascertained graphically in final models via examination of the log-minus-log plot of survival function, and the Schoenfield residuals.

We examined the associations between clusters, disorders and symptom dimensions with future MACCE in three respective models. Model 1 was comprised of covariates and the diagnostic categories of prevalent affective disorders (GAD, panic disorder and major depression). Model 2 was comprised of covariates and the affective disorders were arranged into distress and fear clusters. Model 3 concerned the symptom dimensions of anhedonia, anxious arousal and general NA as measured with the MASQ.

## Results

### Descriptive characteristics

The final sample consisted of 158 CABG patients between 36 and 87 years (mean age = 64.7 years ± 10.6, 20.9 % women, 11.4 % concomitant valve surgery). Baseline characteristics stratified by MACCE are shown in Table 1. Patients experiencing a MACCE were significantly older and were characterized by a higher proportion of hypertension, heart failure and peripheral vascular disease. In the total sample, there were 698 cumulative person years of survival for analysis with a median follow-up of 4.6 years (interquartile range 4.2 to 5.2). There were 37 MACCE events (23.4 % of total), most commonly deaths due to CHD (n = 15), and non-fatal myocardial infarction (n = 13), incident heart failure (n = 5) and stroke (n = 4).

**Table 1:** Baseline characteristics of patients with and without a MACCE after CABG surgery

Descriptive variables	Total N (%)^a^	No MACCE (n = 121)	MACCE (n = 37)	*P*
Age, M ± SD	64.7 ± 10.6	63.7 ± 10.5	67.8 ± 10.4	.04
Female	33 (20.9)	26 (21.5)	7 (18.9)	.74
Aboriginal	6 (3.8)	3 (2.5)	3 (8.1)	.14
BMI, M ± SD	29.1 ± 5.2	29.3 ± 5.0	28.6 ± 5.6	.47
Concomitant valvular procedure	18 (11.4)	12 (9.9)	6 (16.2)	.29
Urgent surgery	34 (21.5)	28 (23.1)	6 (16.2)	.37
Previous MI <30 days	51 (32.3)	39 (32.2)	12 (32.4)	.98
LVEF 45 – 60 %	33 (20.9)	22 (18.2)	11 (29.7)	.39
30 – 45 %	12 (7.4)	9 (7.4)	3 (8.1)	
<30 %	6 (3.3)	4 (3.3)	2 (5.4)	
Hypertension	102 (64.6)	73 (60.3)	29 (78.4)	.04
Hypercholesterolemia	118 (74.7)	91 (75.2)	27 (73.0)	.79
Diabetes, Type 1	2 (1.3)	2 (1.7)	-	.63
Type 2	48 (30.4)	38 (31.4)	10 (27.0)	
Chronic lung disease	33 (20.9)	27 (22.3)	6 (16.2)	.43
Renal disease	11 (7.0)	6 (5.0)	5 (13.5)	.07
Heart failure	40 (25.3)	26 (21.5)	14 (37.8)	.04
Peripheral vascular disease	18 (11.4)	10 (8.3)	8 (21.6)	.03
Cerebrovascular disease	16 (10.1)	12 (9.9)	4 (10.8)	.88
Tobacco smoking	94 (59.5)	73 (60.3)	21 (56.8)	.70
SSRI	6 (3.8)	4 (3.3)	2 (5.4)	.63
Tricyclic	3 (1.9)	1 (0.8)	2 (5.4)	.14
Aspirin	122 (77.2)	91 (75.2)	31 (83.8)	.28
EuroSCORE, Median (IQR)	2.6 (1.5 – 4.7)	2.5 (1.5 – 4.4)	3.1 (2.0 – 6.6)	.24
Pre-CPB Hb, M ± SD	13.9 ± 1.7	14.0 ± 1.7	13.5 ± 2.0	.17
Minutes spent on CPB Median (IQR)	56.0 (42.8 – 73.0)	57.0 (46.0 – 73.0)	52.0 (36.5 – 74.5)	.30
ICU LOS, median hours (IQR)	25.7 (23 – 47.5)	25.7 (23.0 – 48.4)	25.7 (23.6 – 27.6)	.97
ICU intubation, median hours (IQR)	12.5 (10.1 – 17.0)	12.2 (9.6 – 16.4)	14.2 (11.5 – 22.0)	.13

^a^Data presented as N (%) unless otherwise specified

*BMI* body mass index, *CABG* coronary artery bypass graft surgery, *CPB* cardiopulmonary bypass, *ICU* intensive care unit, *IQR* interquartile range, *LVEF* left ventricular ejection fraction, *LOS* length of stay, *M ± SD* mean ± standard deviation, *MACCE* major adverse cardiovascular and cerebrovascular events, *MI* myocardial infarction, *SSRI* selective serotonin re-uptake inhibitor

### Prevalence of affective disorders

Diagnostic interview indicated that major depression was most common (n = 27, 17.1 %), followed by panic disorder (n = 12, 10.8 %) and GAD (n = 16, 10.2 %). In total, there were 39 (24.7 %) patients meeting at least one diagnosis of the distress cluster and 21 (13.3 % of total) participants meeting criteria from the fear cluster.

### Risk factors for major adverse cardiovascular events

**Fig. 2:**
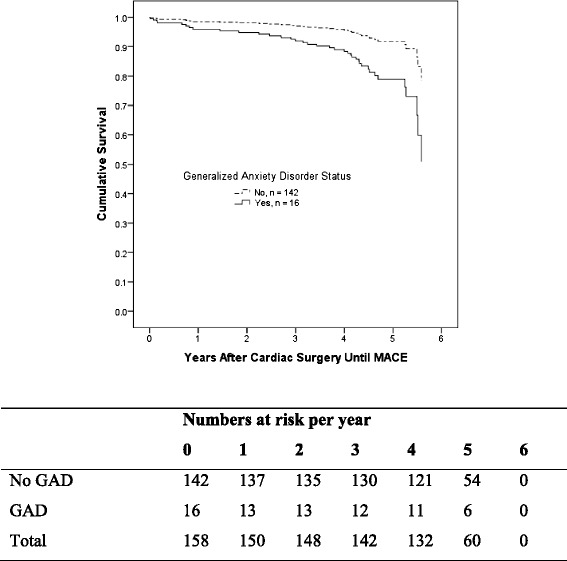
Cox Hazard model survival graph of cumulative survival after CABG surgery until MACCE according to GAD status. Graph showing cumulative survival curves for MACCE comparing patients according to preoperative GAD diagnosis. Dotted line represents no GAD before CABG surgery (n = 142) and the solid black line represents a diagnosis of GAD before CABG surgery (n = 16, adjusted hazard ratio 2.79, 95 % confidence interval 1.00 – 7.80, *p* = .05). Adjustment made for EuroSCORE, smoking, body mass index, hypertension, heart failure, peripheral vascular disease. CABG, coronary artery bypass graft surgery; GAD, generalized anxiety disorder; MACCE, major adverse cardiovascular and cerebrovascular events

Examination of the affective disorders, clusters and symptom dimensions are shown in Table 2. Model 1 suggested that only GAD was significantly associated with an increased risk of MACCE (adjusted HR = 2.79, 95 % CI 1.00 to 7.80, *p* = .05). Fig. 2 depicts the divergence in cumulative survival curves for MACCE in the period after CABG according to GAD status before surgery and is evident within the first year*.* Neither depression nor panic disorder was associated with MACCE (both *p* >. 20).

**Table 2:** Hazard ratios for MACcE after CABG according to affective disorders, disorder clusters, and symptom dimensions

Model structure	N (%)^a^	Hazard ratio^b^	95 % CI lower	95 % CI upper	*P*
*Model 1: Diagnostic Level - Disorders*					
Generalized Anxiety Disorder	16 (10.2)	2.79	1.00	7.80	.049
Major Depression	27 (17.1)	1.04	.40	2.67	.94
Panic Disorder	12 (10.8)	.36	.08	1.76	.21
*Model 2: Theoretical Level - Disorder Clusters*					
Distress disorders^c^	39 (24.7)	2.14	.92	4.95	.08
Fear disorders^d^	21 (13.3)	.24	.05	1.20	.08
*Model 3: Theoretical Level - Symptom Dimensions*					
MASQ General Negative Affect	19.1 ± 3.0	1.07	.89	1.30	.46
MASQ Anhedonia	16.5 ± 3.2	1.04	.85	1.26	.73
MASQ Anxious Arousal	22.8 ± 4.5	.94	.84	1.06	.30

*CABG* coronary artery bypass graft, *CI* confidence interval, *HR* hazard ratio, *MACCE* major adverse cardiovascular and cerebrovascular events, *MASQ* Mood and Anxiety Symptom Questionnaire

^a^The M ± SD is reported for MASQ General Negative Affect, Anhedonia and Anxious Arousal

^b^Hazard model adjusted for EuroSCORE, smoking, body mass index, hypertension, heart failure, peripheral vascular disease

^c^Misery cluster comprised by major depression, dysthymia, generalized anxiety disorder and post-traumatic stress disorder

^d^Fear disorder cluster comprised by panic disorder, agoraphobia, social phobia

[Model 2 shows the association between the distress disorders and MACCE (adjusted HR = 2.14, 95 % CI .92 to 4.95, *p* = .08) and the fear disorders and MACCE (adjusted HR = .24, 95 % CI .05 to 1.20, *p* = .08). Although both associations were above conventional significance, there was a trend for an increased risk for MACE associated with the distress disorders and a reduced risk associated with the fear disorders.

Model 3 shows that anhedonia, anxious arousal and general NA were not associated with MACCE (all *p* >. 30). Covariates significantly associated with MACCE in Models 1 to 3 included heart failure (HR 2.31 to 2.40) and EuroSCORE (HR 1.05 to 1.06).

### Sensitivity analyses

Considering that there was a significant association between GAD and MACCE, but a relatively limited number of MACCE, we performed sensitivity analyses showing the change in effect size attributable to GAD with adjustment for each individual covariate. This analytical strategy involved entering only a single covariate into the GAD model. This approach has been adopted previously [35] and is less prone to overfitting the Cox regression model. The results indicated that a 5 % change in the GAD-MACCE hazard ratio was evident for panic disorder, hypertension and tobacco smoking, though GAD remained significantly associated with MACCE (Table 3). In our second sensitivity analysis, we assessed whether theoretical conceptualizations of depression and anxiety were associated with the acute cardiovascular events of myocardial infarction and cardiac death (combined n = 28). In unadjusted analyses, it was evident that only GAD was related to acute cardiovascular events (unadjusted HR = 2.92, 95 % CI 0.98 to 8.64, *p* = .054).

**Table 3:** Change in the strength of association between generalized anxiety disorder and major adverse cardiovascular events after adjustment for potential confounders

Model covariates	Change in GAD hazard ratio after adjustment, %
Depression disorder	1.6
Panic disorder	5.7
EuroSCORE	3.3
Heart failure	4.2
Hypertension	−8.3
Peripheral vascular disease	−0.3
Body mass index	1.5
Smoking	7.1

GAD, generalized anxiety disorder

## Discussion

This study was the first to comprehensively examine the MACCE risk attributable to affective disorders at the diagnostic, cluster and symptom dimension level in a cohort of CHD patients undergoing CABG surgery. At the diagnostic level, it was evident that GAD was significantly associated with MACCE after cardiac surgery, extending previous findings among CHD out-patients [33, 35]. With respect to the arrangement of disorders into clusters, it was evident that the distress disorders were not significantly associated with increased MACCE risk, and therefore not supporting the hypothesis. The symptom dimensions anhedonia and anxious arousal were not significantly associated with MACCE, thus not supporting the hypothesis nor previous work [30, 31].

The study showed no significant association between MACCE and the distress or fear disorders, their symptom dimensions, panic disorder or major depression. Nonetheless, the GAD findings align with some larger studies [33, 35], albeit contrasting to Parker and colleagues’ cohort [34]. Previously in a large epidemiological survey, Goodwin and colleagues [60] showed that GAD was most strongly associated with lower CHD risk in cross-sectional analyses whereas the mood disorders were not. Potential mechanisms underlying the association with CHD include GAD patients’ propensity toward diminished heart rate variability [61], elevated heart rate, smoking, and hypertension [62], parallel to what has been reported amongst depressed CHD and CABG surgery patients [63–66]. Our arrangement of disorders into the distress cluster, inclusive of GAD and depression, did not show a significant association with MACCE (*p* = .08). However, this finding was possibly limited by the infrequent occurrence of other disorders especially dysthymia and post-traumatic stress disorder, considering that an emerging literature documents an association between these disorders and MACCE in CHD populations [25, 27, 67].

When symptom dimensions were examined, there was no significant association between MASQ anhedonia, anxious arousal or general NA with subsequent MACCE. The findings with respect to anhedonia contrast to Denollet and colleagues study [28] which reported that a four-item anhedonia measure was associated with MACCE in percutaneous coronary intervention patients. Also, Leroy and colleagues’ study [31] showed that the Chapman Physical Anhedonia Scale was associated with twofold increase in MACCE at 3-year follow-up after acute coronary syndrome. Further assessment of the anhedonia symptom dimension may provide insight as to specific underlying mechanisms linking depression and MACCE. For example, the anhedonia symptom dimension is highly associated with biological mechanisms of cardiopathogenesis such as the hypothalamus-pituitary-adrenal axis [68] and the metabolic syndrome [69]. Moreover, symptom dimensions are also associated with other risk factors for cardiopathogenesis such as exposure to childhood trauma, social disadvantage, and adversity in adult life [70].

The current findings may have clinical relevance for the population with GAD who are facing CABG surgery, or comorbid CHD and anxiety disorders generally [71]. Specifically, the findings raise the possibility that psychological interventions targeting GAD are warranted in CABG surgery patients. Recently it was shown that collaborative care programs were effective for a reduction in generalized anxiety and depression symptoms [72, 73]. Moreover, exercise, anxiolytic use and cognitive behavioral therapy for GAD were associated with a reduction in somatic depressive symptoms among persons with comorbid depression disorder-GAD and heart failure [74]. In the population undergoing CABG surgery, prior interventions have indicated small to medium treatment effect sizes regarding reduction in depression and anxiety symptoms [75–78]. Moreover, non-pharmacological intervention is especially advantageous in the CABG population given that selective serotonin re-uptake inhibitors may pose a morbidity risk particularly relating to postoperative hemorrhage [79–81].

This study’s main methodological strength is the delineation of disorders at the diagnostic and cluster level and the measurement of symptom dimensions. There are several limitations to the generalizability of these results including that the psychiatric assessment occurred before CABG surgery. It cannot be ruled out that some persons experienced a worsening of their mental health postoperatively and possibly developed psychiatric comorbidities not quantified in this study. Furthermore, we were not able to identify persons receiving treatment for mental disorders after CABG surgery which may affect the occurrence of MACCE [82]. It is also possible too that anxiety levels were especially higher than normal in the CABG patients during the pre-operative period. With respect to psychiatric comorbidity that is common in persons with depression, we cannot extend our findings to the externalizing cluster of disorders [24], as patients with alcohol and substance abuse were excluded. Also, low base rates were observed for some disorders. Moreover, hierarchical exclusion rules stipulated by the MINI preclude comorbidity between these disorders and would inevitably result in lower depression and GAD prevalence. Consequently, analysis of cardiac morbidity was constrained to a combined MACCE endpoint with adjustment for a limited number of covariates. Indeed, the width of the confidence intervals suggests that future studies may benefit from larger samples. Finally, the participation rate was under-represented by Indigenous Australian peoples partly because persons living in rural and remote areas without a fixed residential address were excluded. As lower access to medical services may disadvantage Indigenous Australian peoples with CHD [83], the findings may not generalize to these cultural groups.

In conclusion, analysis with various theoretical conceptualizations of negative emotions suggested that only GAD was significantly associated with MACCE after CABG surgery. The non-significant association between MACCE and the distress-cluster may warrant further investigation in larger samples. Further research concerning combinations of disorders and negative emotions may contribute to clinical intervention in the population with CHD.

### Informed consent

All procedures followed were in accordance with the ethical standards of the responsible committee on human experimentation (institutional and national) and with the Helsinki Declaration of 1975, as revised in 2000. Informed consent was obtained from all patients for being included in the study.

## Abbreviations

CABG: Coronary artery bypass graft
CHD: Coronary heart disease
CI: Confidence interval
GAD: Generalized anxiety disorder
HR: Hazard ratio
MACCE: Major adverse cardiovascular and cerebrovascular events
MASQ: Mood and anxiety symptom questionnaire
MINI: MINI International Psychiatric Interview
NA: Negative affect

## References

[1] Carney RM, Freedland KE, Sheline YI, Weiss ES (1997). Depression and coronary heart disease: a review for cardiologists. Clin Cardiol.

[2] Dickens C, Cherrington A, Adeyemi I, Roughley K, Bower P, Garrett C (2013). Characteristics of psychological interventions that improve depression in people with coronary heart disease: a systematic review and meta-regression. Psychosom Med.

[3] Baumeister H, Hutter N, Bengel J (2011). Psychological and pharmacological interventions for depression in patients with coronary artery disease. Coch Data Syst Rev.

[4] Linden W (2013). How many meta-analyses does it take to settle a question?. Psychosom Med.

[5] Dreher H (2004). Psychosocial factors in heart disease: a process model. Adv Mind Body Med.

[6] Ladwig KH, Lederbogen F, Albus C, Angermann C, Borggrefe M, Fischer D (2014). Position paper on the importance of psychosocial factors in cardiology: Update 2013. Ger Med Sci.

[7] Pogosova N, Saner H, Pedersen SS, Cupples ME, McGee H, Höfer S, et al. Psychosocial aspects in cardiac rehabilitation: From theory to practice. A position paper from the Cardiac Rehabilitation Section of the European Association of Cardiovascular Prevention and Rehabilitation of the European Society of Cardiology. Eur J Prev Cardiol. in press. 28294802

[8] Hori R, Hayano J, Kimura K, Shibata N, Kobayashi F (2015). Psychosocial factors are preventive against coronary events in Japanese men with coronary artery disease: The Eastern Collaborative Group Study 7.7-year follow-up experience. Biopsychosoc Med.

[9] Baumeister H, Haschke A, Munzinger M, Hutter N, Tully PJ. Inpatient and outpatient costs in patients with coronary artery disease and mental disorders: a systematic review. Biopsychosoc Med. 2015;9. 10.1186/s13030-015-0039-z PMC4427919 25969694

[10] Watson D (2009). Differentiating the mood and anxiety disorders: a quadripartite model. Annu Rev Clin Psychol.

[11] Watson D (2009). Locating anger in the hierarchical structure of affect: comment on Carver and Harmon-Jones (2009). Psychol Bull.

[12] Suls J, Bunde J (2005). Anger, anxiety, and depression as risk factors for cardiovascular disease: the problems and implications of overlapping affective dispositions. Psychol Bull.

[13] Smith TW (2011). Toward a more systematic, cumulative, and applicable science of personality and health: lessons from type D personality. Psychosom Med.

[14] Suls J, Martin R (2011). Heart disease occurs in a biological, psychological, and social matrix: cardiac risk factors, symptom presentation, and recovery as illustrative examples. Ann Behav Med.

[15] Grande G, Romppel M, Barth J (2012). Association between type D personality and prognosis in patients with cardiovascular diseases: a systematic review and meta-analysis. Ann Behav Med.

[16] Kubzansky LD, Cole SR, Kawachi I, Vokonas P, Sparrow D (2006). Shared and unique contributions of anger, anxiety, and depression to coronary heart disease: a prospective study in the normative aging study. Ann Behav Med.

[17] Denollet J, Schiffer AA, Spek V (2010). A general propensity to psychological distress affects cardiovascular outcomes: evidence from research on the type D (distressed) personality profile. Circ: Cardiovasc Qual Outcomes.

[18] Meyer FA, von Känel R, Saner H, Schmid JP, Stauber S. Positive affect moderates the effect of negative affect on cardiovascular disease-related hospitalizations and all-cause mortality after cardiac rehabilitation. Eur J Prev Cardiol. in press. 10.1177/2047487314549745 25208905

[19] Tully PJ, Baune BT (2014). Comorbid anxiety disorders alter the association between cardiovascular diseases and depression: the German National Health Interview and Examination Survey. Soc Psych Psychiatr Epidemiol.

[20] Andrews G, Goldberg DP, Krueger RF, Carpenter WT, Hyman SE, Sachdev P (2009). Exploring the feasibility of a meta-structure for DSM-V and ICD-11: could it improve utility and validity?. Psychol Med.

[21] Goldberg DP, Krueger RF, Andrews G, Hobbs MJ. Emotional disorders: cluster 4 of the proposed meta-structure for DSM-V and ICD-11. Psychol Med. 2011;2043–2059. 10.1017/S0033291709990298 19796429

[22] Eaton NR, Krueger RF, Markon KE, Keyes KM, Skodol AE, Wall M (2013). The structure and predictive validity of the internalizing disorders. J Abnorm Psychol.

[23] Wright AG, Krueger RF, Hobbs MJ, Markon KE, Eaton NR, Slade T (2013). The structure of psychopathology: toward an expanded quantitative empirical model. J Abnorm Psychol.

[24] Krueger RF (1999). The structure of common mental disorders. Arch Gen Psychiatry.

[25] Baune BT, Adrian I, Arolt V, Berger K (2006). Associations between major depression, bipolar disorders, dysthymia and cardiovascular diseases in the general adult population. Psychother Psychosom.

[26] Stenman M, Holzmann MJ, Sartipy U (2014). Relation of major depression to survival after coronary artery bypass grafting. Am J Cardiol.

[27] Rafanelli C, Roncuzzi R, Milaneschi Y (2006). Minor depression as a cardiac risk factor after coronary artery bypass surgery. Psychosomatics.

[28] Denollet J, Pedersen SS, Daemen J, de Jaegere P, Serruys PW, van Domburg RT (2008). Reduced positive affect (anhedonia) predicts major clinical events following implantation of coronary-artery stents. J Intern Med.

[29] Pelle AJ, Pedersen SS, Szabo BM, Denollet J (2009). Beyond Type D personality: reduced positive affect (anhedonia) predicts impaired health status in chronic heart failure. Qual Life Res.

[30] Davidson KW, Burg MM, Kronish IM, Shimbo D, Dettenborn L, Mehran R (2010). Association of anhedonia with recurrent major adverse cardiac events and mortality 1 year after acute coronary syndrome. Arch Gen Psychiatry.

[31] Leroy M, Loas G, Perez-Diaz F (2010). Anhedonia as predictor of clinical events after acute coronary syndromes: a 3-year prospective study. Compr Psychiatry.

[32] Edmondson D, Rieckmann N, Shaffer JA, Schwartz JE, Burg MM, Davidson KW (2011). Posttraumatic stress due to an acute coronary syndrome increases risk of 42-month major adverse cardiac events and all-cause mortality. J Psychiatr Res.

[33] Frasure-Smith N, Lespérance F (2008). Depression and anxiety as predictors of 2-year cardiac events in patients with stable coronary artery disease. Arch Gen Psychiatry.

[34] Parker G, Hyett M, Hadzi-Pavlovic D, Brotchie H, Walsh W (2011). GAD is good? Generalized anxiety disorder predicts a superior five-year outcome following an acute coronary syndrome. Psychiatry Res.

[35] Martens EJ, de Jonge P, Na B, Cohen BE, Lett H, Whooley MA (2010). Scared to death? Generalized anxiety disorder and cardiovascular events in patients with stable coronary heart disease: The Heart and Soul Study. Arch Gen Psychiatry.

[36] Tully PJ, Cosh SM, Baumeister H (2014). The anxious heart in whose mind? A systematic review and meta-regression of factors associated with anxiety disorder diagnosis, treatment and morbidity risk in coronary heart disease. J Psychosom Res.

[37] Tully PJ, Pedersen SS, Winefield HR, Baker RA, Turnbull DA, Denollet J (2011). Cardiac morbidity risk and depression and anxiety: a disorder, symptom and trait analysis among cardiac surgery patients. Psychol Health Med.

[38] Tully PJ, Newland RF, Baker RA (2015). Cardiovascular risk profile before coronary artery bypass graft surgery in relation to depression and anxiety disorders: an age and sex propensity matched study. Aust Crit Care.

[39] The Australian Society of Cardiac and Thoracic Surgery (2008). ASCTS cardiac surgery database project: data definitions.

[40] Sheehan DV, Lecrubier Y, Harnett-Sheehan K, Janavs J, Weiller E, Bonora LI (1997). Reliability and Validity of the MINI International Neuropsychiatric Interview (M.I.N.I.): according to the SCID-P. Eur Psychiatry.

[41] Lecrubier Y, Sheehan DV, Weiller E, Amorim P, Bonora I, Sheehan KH (1997). The MINI International Neuropsychiatric Interview (M.I.N.I.). A short diagnostic structured interview: reliability and validity according to the CIDI. Eur Psychiatry.

[42] van Vliet IM, de Beurs E (2007). The MINI-International Neuropsychiatric Interview. A brief structured diagnostic psychiatric interview for DSM-IV en ICD-10 psychiatric disorders. Tijdschr Psychiatr.

[43] Watson D, Weber K, Assenheimer JS, Clark LA, Strauss ME, McCormick RA (1995). Testing a tripartite model: I. Evaluating the convergent and discriminant validity of anxiety and depression symptom scales. J Abnorm Psychol.

[44] Wardenaar KJ, van Veen T, Giltay EJ, de Beurs E, Penninx BW, Zitman FG (2010). Development and validation of a 30-item short adaptation of the Mood and Anxiety Symptoms Questionnaire (MASQ). Psychiatry Res.

[45] Tully PJ, Wardenaar KJ, Penninx BW (2015). Operating characteristics of depression and anxiety disorder phenotype dimensions and trait neuroticism: a theoretical examination of the fear and distress disorders from the Netherlands study of depression and anxiety. J Affect Disord.

[46] Lin A, Yung AR, Wigman JT, Killackey E, Baksheev G, Wardenaar KJ (2014). Validation of a short adaptation of the Mood and Anxiety Symptoms Questionnaire (MASQ) in adolescents and young adults. Psychiatry Res.

[47] Reidy J, Keogh E (1997). Testing the discriminant and convergent validity of the mood and anxiety sympotms questionnaire using a British sample. Personal Individ Differ.

[48] Keogh E, Reidy J (2000). Exploring the factor structure of the Mood and Anxiety Symptom Questionnaire (MASQ). J Pers Assess.

[49] Bedford A, Lukic G, Allerhand M, Deary IJ (2011). Mood and anxiety symptom questionnaire anxiety items in an adult British clinical sample: one scale or two?. Clin Psychol Psychother.

[50] Schalet BD, Cook KF, Choi SW, Cella D (2014). Establishing a common metric for self-reported anxiety: linking the MASQ, PANAS, and GAD-7 to PROMIS Anxiety. J Anxiety Disord.

[51] de Beurs E, den Hollander-Gijsman ME, Helmich S, Zitman FG (2007). The tripartite model for assessing symptoms of anxiety and depression: psychometrics of the Dutch version of the mood and anxiety symptoms questionnaire. Behav Res Ther.

[52] Nitschke JB, Heller W, Imig JC, McDonald RP, Miller GA (2001). Distinguishing dimensions of anxiety and depression. Cogn Ther Res.

[53] World Health Organization. International Statistical Classification of Diseases and Related Health Problems 10th Revision Version for 2007 Available online at http://www.who.int/classifications/apps/icd/icd10online/. 2007.

[54] de Jonge P, Ormel J, van den Brink RH, van Melle JP, Spijkerman TA, Kuijper A (2006). Symptom dimensions of depression following myocardial infarction and their relationship with somatic health status and cardiovascular prognosis. Am J Psychiatr.

[55] Rothman KJ (1990). No adjustments are needed for multiple comparisons. Epidemiology.

[56] Almeida OP, Alfonso H, Pirkis J, Kerse N, Sim M, Flicker L (2011). A practical approach to assess depression risk and to guide risk reduction strategies in later life. Int Psychogeriatr.

[57] Biancari F, Kangasniemi OP, Luukkonen J, Vuorisalo S, Satta J, Pokela R (2006). EuroSCORE predicts immediate and late outcome after coronary artery bypass surgery. Ann Thorac Surg.

[58] Kobayashi KJ, Williams JA, Nwakanma LU, Weiss ES, Gott VL, Baumgartner WA (2009). EuroSCORE predicts short- and mid-term mortality in combined aortic valve replacement and coronary artery bypass patients. J Card Surg.

[59] Nashef SA, Roques F, Michel P, Gauducheau E, Lemeshow S, Salamon R (1999). European system for cardiac operative risk evaluation (EuroSCORE). Eur J Cardiothorac Surg.

[60] Goodwin RD, Davidson KW, Keyes K (2009). Mental disorders and cardiovascular disease among adults in the United States. J Psychosom Res.

[61] Chalmers J, Quintana DS, Abbott MJ, Kemp AH. Anxiety disorders are associated with reduced heart rate variability: a meta-analysis. Front Psychiatry. 2014;5. 10.3389/fpsyt.2014.00080 PMC4092363 25071612

[62] Tully PJ, Cosh SM, Baune BT (2013). A review of the affects of worry and generalized anxiety disorder upon cardiovascular health and coronary heart disease. Psychol Health Med.

[63] Shaffer JA, Whang W, Shimbo D, Burg M, Schwartz JE, Davidson KW (2012). Do different depression phenotypes have different risks for recurrent coronary heart disease?. Health Psychol Rev.

[64] Patron E, Messerotti Benvenuti S, Favretto G, Valfrè C, Bonfà C, Gasparotto R (2012). Association between depression and heart rate variability in patients after cardiac surgery: a pilot study. J Psychosom Res.

[65] Patron E, Messerotti Benvenuti S, Zanatta P, Polesel E, Palomba D (2013). Preexisting depressive symptoms are associated with long-term cognitive decline in patients after cardiac surgery. Gen Hosp Psychiatry.

[66] Dao TK, Youssef NA, Gopaldas RR, Chu D, Bakaeen F, Wear E (2010). Autonomic cardiovascular dysregulation as a potential mechanism underlying depression and coronary artery bypass grafting surgery outcomes. J Cardiothorac Surg.

[67] Edmondson D, Richardson S, Falzon L, Davidson KW, Mills MA, Neria Y (2012). Posttraumatic stress disorder prevalence and risk of recurrence in acute coronary syndrome patients: a meta-analytic review. PLoS ONE.

[68] Wardenaar KJ, Vreeburg SA, van Veen T, Giltay EJ, Veen G, Penninx BW (2011). Dimensions of depression and anxiety and the hypothalamo-pituitary-adrenal axis. Biol Psychiatry.

[69] Luppino FS, van Reedt Dortland AK, Wardenaar KJ, Bouvy PF, Giltay EJ, Zitman FG (2011). Symptom dimensions of depression and anxiety and the metabolic syndrome. Psychosom Med.

[70] van Veen T, Wardenaar KJ, Carlier IV, Spinhoven P, Penninx BW, Zitman FG (2013). Are childhood and adult life adversities differentially associated with specific symptom dimensions of depression and anxiety? Testing the tripartite model. J Affect Disord.

[71] Rollman BL, Huffman JC (2013). Treating anxiety in the presence of medical comorbidity: calmly moving forward. Psychosom Med.

[72] Huffman JC, Mastromauro CA, Beach SR, Celano CM, Dubois CM, Healy BC (2014). Collaborative care for depression and anxiety disorders in patients with recent cardiac events: The Management of Sadness and Anxiety in Cardiology (MOSAIC) randomized clinical trial. JAMA Intern Med.

[73] Huffman JC, Mastromauro CA, Sowden GL, Wittmann C, Rodman R, Januzzi JL (2011). A collaborative care depression management program for cardiac inpatients: depression characteristics and in-hospital outcomes. Psychosomatics.

[74] Tully PJ, Selkow T, Bengel J, Rafanelli C (2015). A dynamic view of comorbid depression and generalized anxiety disorder symptom change in chronic heart failure: discrete effects of cognitive behavioral therapy, exercise rehabilitation, and psychotropic medication. Disabil Rehabil.

[75] Dao TK, Youssef NA, Armsworth M, Wear E, Papathopoulos KN, Gopaldas R (2011). Randomized controlled trial of brief cognitive behavioral intervention for depression and anxiety symptoms preoperatively in patients undergoing coronary artery bypass graft surgery. J Thorac Cardiovasc Surg.

[76] Doering LV, Chen B, Cross Bodan R, Magsarili MC, Nyamathi A, Irwin MR (2013). Early cognitive behavioral therapy for depression after cardiac surgery. J Cardiovasc Nurs.

[77] Freedland KE, Skala JA, Carney RM, Rubin EH, Lustman PJ, Davila-Roman VG (2009). Treatment of depression after coronary artery bypass surgery: a randomized controlled trial. Arch Gen Psychiatry.

[78] Rollman BL, Belnap BH, LeMenager MS, Mazumdar S, Schulberg HC, Reynolds CF (2009). The Bypassing the Blues treatment protocol: stepped collaborative care for treating post-CABG depression. Psychosom Med.

[79] Hackam DG, Mrkobrada M (2012). Selective serotonin reuptake inhibitors and brain hemorrhage: a meta-analysis. Neurology.

[80] Auerbach AD, Vittinghoff E, Maselli J, Pekow PS, Young JQ, Lindenauer PK (2013). Perioperative use of selective serotonin reuptake inhibitors and risks for adverse outcomes of surgery. JAMA Intern Med.

[81] Tully PJ, Cardinal T, Bennetts JS, Baker RA (2012). Selective serotonin reuptake inhibitors, venlafaxine and duloxetine are associated with in hospital morbidity but not bleeding or late mortality after coronary artery bypass graft surgery. Heart Lung Circ.

[82] Rollman BL, Belnap BH, LeMenager MS, Mazumdar S, Houck PR, Counihan PJ (2009). Telephone-delivered collaborative care for treating post-CABG depression: a randomized controlled trial. J Am Med Assoc.

[83] Prabhu A, Tully PJ, Bennetts JS, Tuble SC, Baker RA (2013). The morbidity and mortality outcomes of indigenous Australian peoples after isolated coronary artery bypass graft surgery: the influence of geographic remoteness. Heart Lung Circ.

